# A nomogram based on TFE3 IHC results and clinical factors as a preliminary screening scheme for TFE3‐rearranged renal cell carcinoma

**DOI:** 10.1002/cam4.6813

**Published:** 2024-03-13

**Authors:** Pengju Li, Quanhui Xu, Minyu Chen, Jiangquan Zhu, Yinghan Wang, Mukhtar A. Mumin, Kangbo Huang, Zeying Jiang, Hui Liang, Qiong Deng, Zhu Wang, Bing Liao, Wenfang Chen, Yun Cao, Jiazheng Cao, Junhang Luo

**Affiliations:** ^1^ Department of Urology The First Affiliated Hospital of Sun Yat‐sen University Guangzhou China; ^2^ Department of Urology The Cancer Center of Sun Yat‐sen University Guangzhou China; ^3^ Department of Pathology The First Affiliated Hospital of Sun Yat‐sen University Guangzhou China; ^4^ Department of Urology Affiliated Longhua People's Hospital, Southern Medical University Shenzhen China; ^5^ Department of Pathology The Cancer Center of Sun Yat‐sen University Guangzhou China; ^6^ Department of Urology Jiangmen Central Hospital Jiangmen China; ^7^ Institute of Precision Medicine, The First Affiliated Hospital, Sun Yat‐sen University Guangzhou China

**Keywords:** diagnosis, fluorescence in situ hybridization, immunohistochemistry, TFE3‐rearranged renal cell carcinoma

## Abstract

**Background:**

TFE3 immunohistochemistry (TFE3‐IHC) is controversial in the diagnosis of TFE3‐rearranged renal cell carcinoma (TFE3‐rearranged RCC). This study is to investigate the accuracy and sensitivity of IHC and establish a predictive model to diagnose TFE3‐rearranged RCC.

**Methods:**

Retrospective analysis was performed by collecting IHC and fluorescence in situ hybridization (FISH) results from 228 patients. IHC results were evaluated using three scoring systems. Scoring system 1 is graded based on nuclear staining intensity, scoring system 2 is graded based on the percentage of stained tumor cell nuclei, and scoring system 3 is graded based on both the nuclear staining intensity and the percentage. We collected patients' IHC results and clinical information. Important variables were screened based on univariate logistic regression analysis. Then, independent risk factors were established through multivariate logistic regression, and a nomogram model was constructed. The model was validated in internal test set and external validation set. The receiver operating characteristic curve (ROC curve), calibration curve, and decision curve analysis (DCA) were generated to assess discriminative ability of the model.

**Results:**

The accuracy of IHC based on three scoring systems were 0.829, 0.772, and 0.807, respectively. The model included four factors including age, gender, lymph node metastasis and IHC results. Area under the curve (AUC) values were 0.935 for the training set, 0.934 for the internal test set, 0.933 for all 228 patients, and 0.916 for the external validation set.

**Conclusions:**

TFE3 IHC has high accuracy in the diagnosis of TFE3‐rearranged RCC. Clinical information such as age and lymph node metastasis are independent risk factors, which can be used as a supplement to the results of TFE3 IHC. This study confirms the value of IHC in the diagnosis of TFE3‐rearranged RCC. The accuracy of the diagnosis can be improved by incorporating IHC with other clinical risk factors.

## 1 INTRODUCTION

TFE3‐rearranged renal cell carcinoma (RCC) is a rare renal carcinoma characterized by a fusion of TFE3 genes caused by ectopic Xp11.2, resulting in the activation of TFE3.^1^ In 2004, WHO classified Xp11.2/TFE3 translocation RCC as an independent subtype of renal carcinoma, and in 2016, it was reclassified as microphthalmia‐associated transcription factor (MiTF) RCC subtype.^2^ MiTF RCC accounts for about 40% of all children's RCC, while this figure is about 1%–4% in adults. TFE3‐translocation RCC is the most common of all MiTF RCCs.^3^ In 2022, it was reclassified by WHO as TFE3‐rearranged RCC.^4^ TFE3‐rearranged RCC is a clinically aggressive malignant tumor. Compared with ordinary papillary renal carcinoma patients, cancer‐specific survival (CSS) for TFE3‐rearranged RCC is significantly poorer.^5^ Histologically, TFE3‐rearranged RCC is difficult to differentiate from other renal carcinoma subtypes. The positive expression of TFE3 was initially considered to be able to differentiate this type of RCC.^6^ TFE3 protein is widely expressed in human tissues; there are many reasons that will affect the IHC results.^7^ With the deepening of research, researchers found that TFE3 IHC often showed false negative or false positive results. In consideration of this, the detection of Xp11.2 gene rearrangement by FISH is now the gold standard for the diagnosis of TFE3‐rearranged RCC.^8^

At present, the diagnosis process based on TFE3 IHC results seems to be questionable. In order to reveal the role of TFE3 IHC in the diagnosis of TFE3‐rearranged RCC, we have collected the clinical information and pathological specimens of RCC under the age of 30 in the First Affiliated Hospital of Sun Yat‐sen University and the Cancer Center of Sun Yat‐sen University from 2010 to 2021 to further explore the accuracy and sensitivity of IHC as a preliminary screening for TFE3‐rearranged RCC. In addition, we hope to identify other independent risk factors to establish a risk model to predict and diagnose TFE3‐rearranged RCC.

## 2 METHODS

### 2.1 Clinical data and pathological specimens of patients

In pathological practice, FISH can detect translocations using break‐apart probes for the TFE3 and TFEB genes, and it is recommended for diagnosing renal cell carcinoma in patients under 30 years old.^9^ In our study, we first excluded patients with nonrenal cell carcinoma based on pathological results; for instance, patients with Wilms' tumor or other kidney tumors were not included in the analysis. We collected a total of 228 patients under the age of 30 from the First Affiliated Hospital of Sun Yat‐sen University and the Cancer Center Affiliated to Sun Yat‐sen University from 2010 to 2021 and performed TFE3 immunohistochemical staining and FISH detection. We also collected clinical data, which includes general information such as gender and age as well as tumor‐related information such as tumor size, stage, and so on. Lymph node dissection is not a standard procedure during nephrectomy. Therefore, we have to rely on the patient's preoperative radiological data to determine if there is lymph node metastasis. Our assessment is based on both the short‐axis length of the lymph node and the ratio of long to short axis to comprehensively evaluate the presence of lymph node metastasis.^10^

### 2.2 Ethics approval

All specimens were paraffin‐embedded tissue blocks fixed in formalin. This study was conducted in accordance with the ethical standards of the Declaration of Helsinki and was approved by the ethics review committee of the First Affiliated Hospital of Sun Yat‐sen University and the Cancer Center Affiliated to Sun Yat‐sen University, which waived the need of informed consent in ethics approval.

### 2.3 Fluorescence in situ hybridization assay

A dual‐color break‐apart probe (GSP TFE3, Anbiping Company) was used to detect TFE3 rearrangement. All TFE3‐rearranged RCC cases were finally confirmed by FISH assay as previously described.^11^, ^12^ Briefly, 4‐μm‐thick tumor sections were deparaffinized and cooked for 25 min at 100°C in distilled water and then incubated in pepsin solution (4 mg/mL in 0.1 N HCl and 0.9% NaCl) at 37°C for 20 min for digestion. After a 3‐min wash in washing buffer (2× SSC), The sections were dehydrated in ethanol with graded concentrations (70%, 85%, and 100%) for 3 min at room temperature. After drying, probe was added to the tumor region in every slide and co‐denatured with the target DNA at 80 °C for 5 min, followed by hybridization at 37°C overnight in a humidified incubator. Following several washing steps and air drying, the sections were stained with DAPI (Insitus) and get coverslips on.

### 2.4 FISH evaluation

A Zeiss LSM880 microscope (Zeiss) was used to examine sections. A normal result was exhibited as a fusion signal or closely approximated green‐red signal pattern, whereas the TFE3 rearrangement result was exhibited as a split‐signal pattern, which showed that the green and red signals were separated by a distance greater than 2 signal diameters. At least 100 tumor nuclei were examined under microscopy at 1000× magnification for each case. We only evaluated nonoverlapping tumor nuclei. A positive result was reported if at least 10% of the tumor nuclei showed the split‐signal pattern.^13^, ^14^, ^15^, ^16^

### 2.5 Immunohistochemistry

The 4‐μm‐thick tumor sections were deparaffinized in xylene for 30 min and rehydrated using graded ethanol concentrations. Epitope retrieval was performed by a pressure cooker for 2.5 min. After being treated with hydrogen peroxidase blocker (PV‐6001, ZSGB‐BIO), sections were incubated at 4°C overnight with a TFE3 antibody (ZA‐0657, ZSGB‐BIO) and followed with goat anti‐rabbit IgG antibody (PV‐6000, ZSGB‐BIO) for 20 min at room temperature after a washing step. In order to show appropriate immunostaining, positive and negative controls were also stained.

### 2.6 Scoring of TFE3 nuclear immunoreactivity

The results of TFE3 IHC were evaluated in three scoring systems, respectively, listed as follows. Cytoplasmic staining was ignored. Scoring system 1^5^, ^17^, ^18^: The intensity of nuclear immunoreactivity was graded as “negative or 0”, “mild or 1+”, “moderate or 2+”, and “strong or 3+” for all tumor nuclei. Cases were considered to be positive when labeling was moderate (2+) to strong (3+), whether non‐diffuse or diffuse, which was apparent at low‐power magnification (×40 magnification). Cases with focal staining (<10% of the cells) and mild intensity labeling were excluded. Scoring system 2^19^: The percentage of stained tumor nuclei was scored as “negative or 0”, “focal or 1+” (<10% stained nuclei), “moderate or 2+” (10%–50% stained nuclei), and “diffuse or 3+” (>50% stained nuclei). Cases were considered to be positive when labeling was moderate (2+) or diffuse (3+) immunoreactivity. Scoring system 3 was based on both tumor nuclear intensity and the percentage of stained tumor cell nuclei.^20^ The score was calculated by multiplying the nuclear intensity (0 = no staining, 1 = mild staining, 2 = moderate staining, and 3 = strong staining) by the percentage of stained tumor cell nuclei (0–100). Cases showing score of <25 were considered to be “negative or 0”. Cases showing score of 26–100 were considered to be “weak positive or 1+”. Cases showing score of 101–200 were considered to be “moderate positive or 2+”. Cases showing score of 201–300 were considered to be “strong positive or 3+”.

### 2.7 Establishment and validation of the nomogram prediction model

The chi‐square test is used to compare categorical variables. The student t‐test is used to compare the mean values of continuous variables between groups. After selecting important variables through univariate logistic regression analysis, they are included in multivariate logistic regression analysis. If a statistically significant difference is observed in multivariate logistic regression analysis, the variable is considered an independent predictive factor. Based on the final logistic risk regression model, a nomogram model is constructed using the R rms package, and internal and external validations are performed. The ROC curve is used to evaluate the discriminative ability of the model. An AUC value closer to 1 indicates better predictive results of the model, while an AUC value closer to 0.5 indicates that the model does not have predictive capability. The calibration curve is used to assess the consistency of the model, and the DCA curve is used to evaluate the clinical utility of the model.

### 2.8 Statistical methods

All statistics were analyzed by R Studio, and *p* < 0.05 was considered to be statistically significant.

## 3 RESULTS

### 3.1 TFE3 FISH results

A total of 228 cases were included in this study, of which 59 cases were positive determined by FISH, accounting for about 25.9%, and 169 cases were negative, accounting for about 74.1%. When the result is positive, the pathological pictures of male patients show a pair of separated red and green signals, or a single red or green signal due to visual field truncation; Pathological pictures of women showed a pair of separated red and green signals and a normal yellow hybrid signal. The negative results showed one (male) or two (female) normal yellow fusion hybridization signals in male and female patients, respectively (Figure S1).

### 3.2 Patient characteristics

The clinical features of the whole cohort are collected and summarized in Table 1. The mean age at diagnosis of non‐TFE3‐rearranged RCC was 26.1 years, and the median age was 27.0 years. The mean age at diagnosis of TFE3‐rearranged RCC was 19.4 years, with a median age of 22.0 years. The incidence of TFE3‐rearranged RCC (40.7% in men vs. 59.3% in women) was higher in females when compared to other RCC (68.6% in men versus 31.4% in women). In addition, the proportion of advanced tumors, lymph node metastases, and distant metastases was all higher in TFE3‐rearranged RCC. These results suggest that TFE3‐rearranged RCC is more clinically aggressive.

**TABLE 1:** Clinicopathological characteristics.

	Non‐TFE3 (*n* = 169)	TFE3 (*n* = 59)
Age (yr)		
Mean (SD)	26.1 (3.92)	19.4 (7.11)
Median (Min, Max)	27.0 (8.00, 30.0)	22.0 (4.00, 29.0)
Gender, *n* (%)		
Male	116 (68.6)	24 (40.7)
Female	53 (31.4)	35 (59.3)
MTD (cm)		
Mean (SD)	5.16 (3.33)	6.67 (3.57)
Median (Min, Max)	4.30 (1.20, 19.1)	5.85 (2.50, 15.6)
Missing (*n*, %)	3 (1.8)	1 (1.7)
T stage, *n* (%)		
cT1	125 (74.0%)	30 (50.8%)
cT2	30 (17.8%)	6 (10.2%)
cT3	8 (4.7%)	16 (27.1%)
cT4	6 (3.6%)	7 (11.9%)
Node status, *n* (%)		
cN0	158 (93.5%)	36 (61.0%)
cN1	11 (6.5%)	23 (39.0%)
Metastasis status, *n* (%)		
cM0	162 (95.9%)	50 (84.7%)
cM1	7 (4.1%)	9 (15.3%)
Clinical stage, *n* (%)		
I	120 (71.0%)	21 (35.6%)
II	27 (16.0%)	4 (6.8%)
III	12 (7.1%)	21 (35.6%)
IV	10 (5.9%)	13 (22.0%)

Abbreviation: MTD, Maximum tumor diameter.

### 3.3 TFE3 IHC results

To evaluate the role of IHC in the clinical primary screening of TFE3‐rearranged RCC, we assessed it using three scoring criteria based on the available literature (Figure S2). The first scoring system was based on the intensity of TFE3 in nuclear staining. Among them, 129 cases (56.6%) were rated as 0 for TFE3, 31 cases (13.5%) were 1+, 35 cases (15.4%) were 2+, and 33 cases (14.5%) were 3+. The second scoring criterion was based on the proportion of positive nuclear expression of TFE3. Among them, 101 cases (44.3%) were 0 for TFE3, 28 cases (12.3%) were 1+, 46 cases (20.2%) were 2+, and 53 cases (23.2%) were 3+. The third scoring criterion was a semi‐quantitative approach based on the intensity of the markers and the percentage of nuclei in the immunopositive tumors, of which 145 cases (63.6%) were 0 for TFE3, 39 cases (17.1%) were 1+, 24 cases (10.5%) were 2+, and 20 cases (8.8%) were 3+. The final results of the three scoring standards are relatively consistent with the results of FISH (Table 2).

**TABLE 2:** Results of different IHC scoring system.

	Non‐TFE3 (*n* = 169)	TFE3 (*n* = 59)
Scoring system1, *n* (%)		
0	123 (72.8)	6 (10.2)
1+	22 (13.0)	9 (15.3)
2+	19 (11.2)	16 (27.1)
3+	5 (3.0)	28 (47.5)
Scoring system2, *n* (%)		
0	101 (59.8)	0 (0)
1+	22 (13.0)	6 (10.2)
2+	32 (18.9)	14 (23.7)
3+	14 (8.3)	39 (66.1)
Scoring system3, *n* (%)		
0	135 (79.9)	10 (16.9)
1+	25 (14.8)	14 (23.7)
2+	8 (4.7)	16 (27.1)
3+	1 (0.6)	19 (32.2)

### 3.4 Assessment of the effectiveness and accuracy of IHC results

In order to further evaluate the effectiveness and accuracy of IHC as a primary screening method for TFE3‐rearranged RCC, we further analyzed the classification results based on IHC. The first scoring system diagnosed 68 (29.8%) positive cases and 160 (70.2%) negative cases. The second scoring system diagnosed 99 (43.4%) positive cases and 129 (56.6%) negative cases. The third scoring system diagnosed 83 (36.4%) positive cases and 145 (63.6%) negative cases. Compared with the FISH diagnosis, the accuracy of the first scoring system was 0.829, the sensitivity was 0.746, the specificity was 0.858, and 39 cases (17.1%) were missed and misdiagnosed. The accuracy, sensitivity, and specificity of the second scoring system were 0.772, 0.898, and 0.728, respectively, and 52 (22.8%) cases were missed and misdiagnosed. The accuracy, sensitivity, and specificity of the third scoring system were 0.807, 0.831, and 0.799, respectively, and 44 (19.3%) cases were missed and misdiagnosed (Table 3).

**TABLE 3:** Characteristics of confusion matrix based on three scoring system.

	Accuracy	Balanced accuracy	Sensitivity	Specificity	PPV	NPV	F1
Scoring system 1	0.829	0.802	0.746	0.858	0.647	0.906	0.693
Scoring system 2	0.772	0.813	0.898	0.728	0.535	0.954	0.671
Scoring system 3	0.807	0.815	0.831	0.799	0.590	0.931	0.690

Abbreviations: NPV, negative predictive value; PPV, positive predictive value.

### 3.5 Establishment and evaluation of the risk model

**FIGURE 1:**
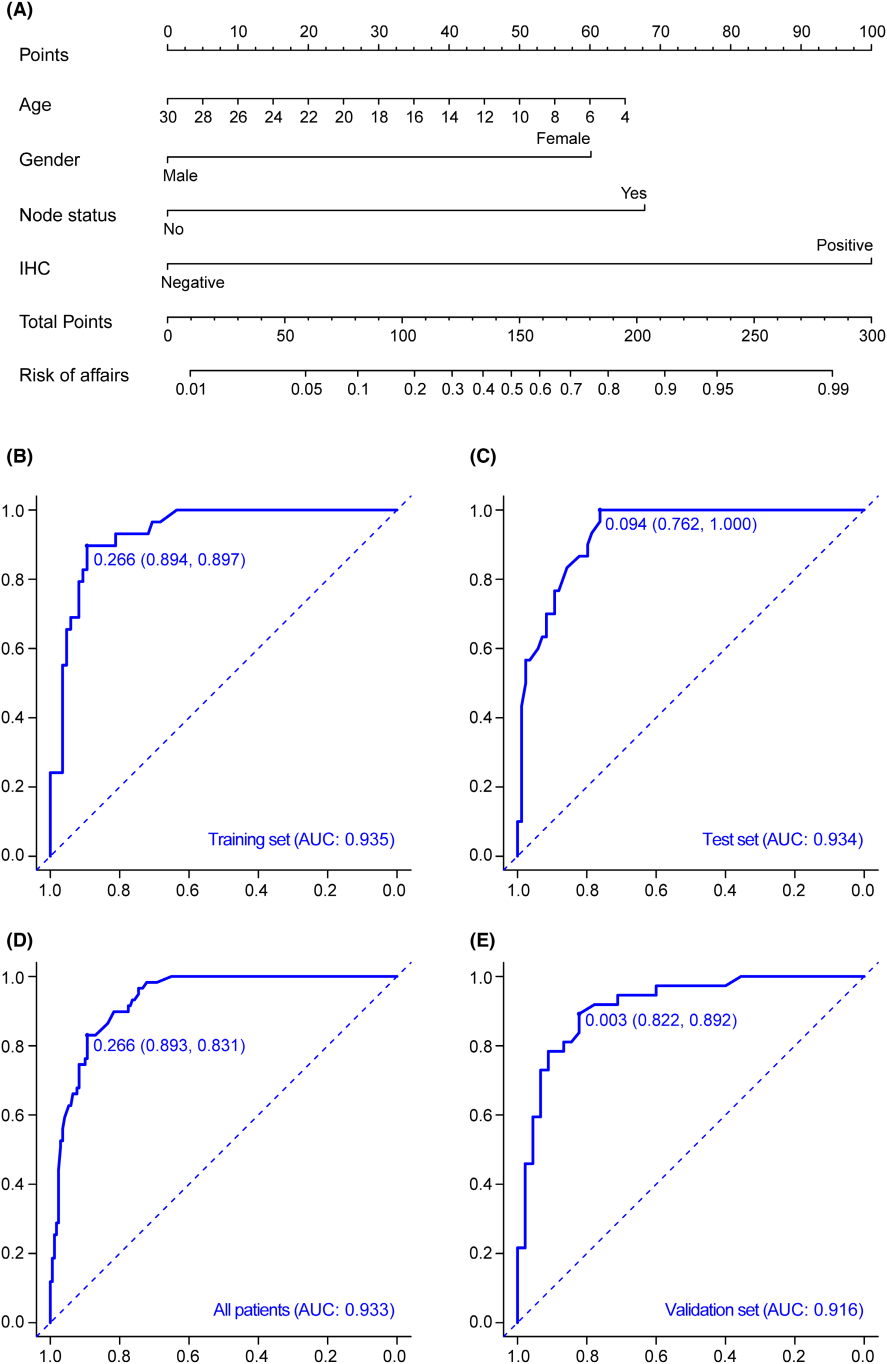
A nomogram to predict TFE3‐rearranged RCC and ROC curves of the nomogram. (A) A nomogram for predicting TFE3‐rearranged RCC (B–E) ROC curves of the nomogram in the training (B), internal test (C), all patients (D) and external validation cohorts (E).

**FIGURE 2:**
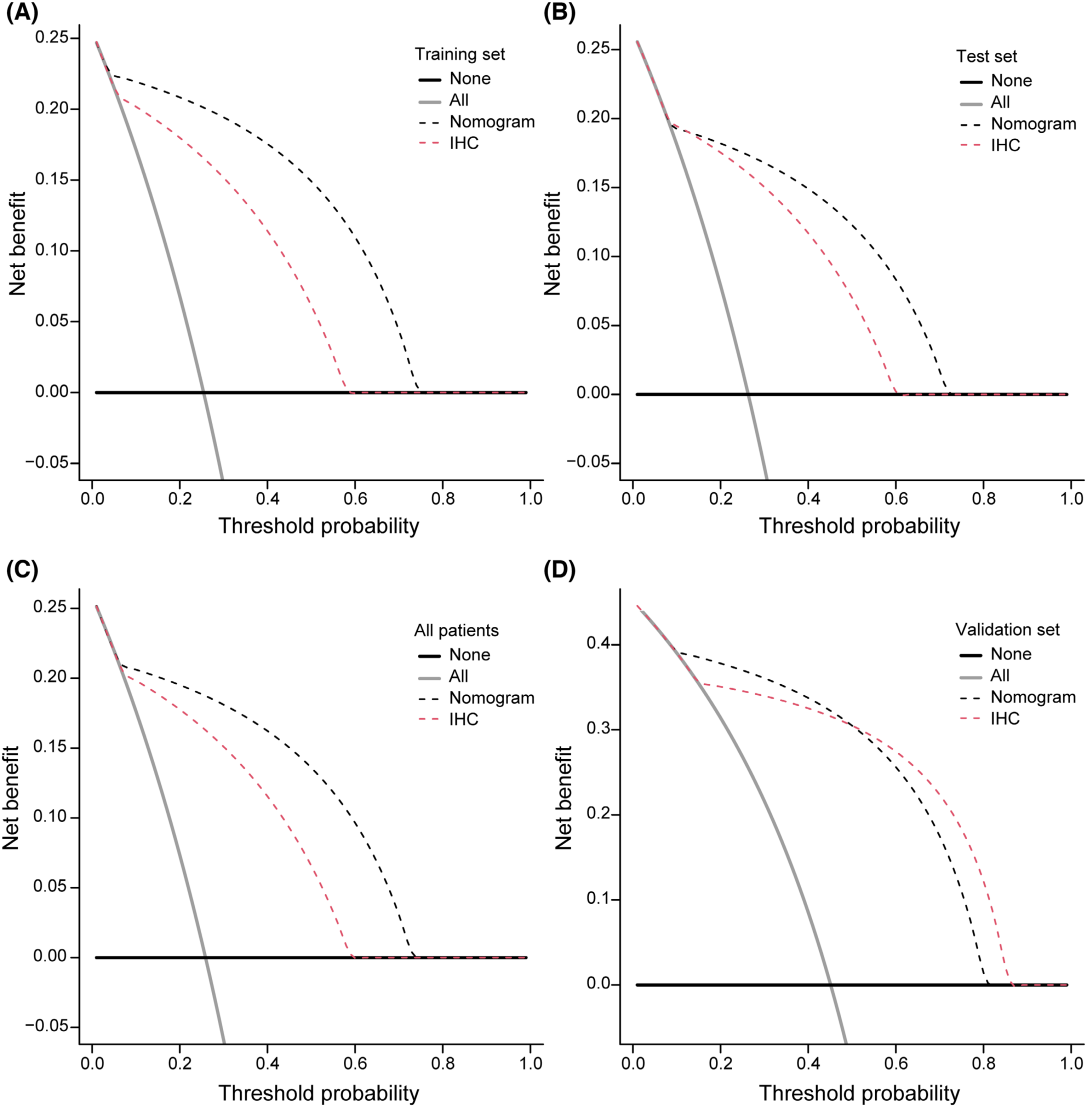
Plots of decision curve results of the nomogram. Decision curve analysis of the nomogram in in the training (A), internal test (B), all patients (C) and external validation cohorts (D).

Multivariate logistic regression analysis suggested that there were 4 independent risk factors, followed by age (OR = 0.86, *p* = 0.001), gender (OR = 0.29, *p* = 0.013), lymph node metastasis (OR = 6.00, *p* = 0.048), and IHC (OR = 21.45, *p* < 0.001) (Table 4). We split the raw data into a training set and an internal test set in a 1:1 ratio and collected 82 cases from previous articles that included the above variables as the external validation set.^11^, ^20^, ^21^, ^22^ On the basis of logistic analysis, we establish a nomogram prediction model, and the final total score can be reflected by the sum of the points of the four factors (Figure 1A). The ROC curve was drawn based on the predicted results and the FISH results, with the area under the curve (AUC) being 0.935 (training set), 0.934 (internal test set), 0.933 (all of the 228 patients), and 0.916 (external validation set) (Figure 1B–E). Calibration results indicated that the model fitted well in all of the data set (Figure S3 **)**. Decision curve analysis was widely used to assess the accuracy of nomogram‐aided decision‐making by estimating the net benefit of the model based on the difference between the number of true positive and false positive results. In our study, analysis of the DCA curve demonstrated the clinical utility of the nomogram (Figure 2).

**TABLE 4:** Univariable and multivariable logistic regression results.

	Univariable		Multivariable	
	OR	*p* Value	OR	*p* Value
Age (yr)	0.81	<0.001	0.86	0.001
Gender (male vs. female)	0.31	<0.001	0.29	0.013
MTD(cm)	1.12	0.005	0.9	0.192
T	2.02	<0.001	1.07	0.884
N	9.18	<0.001	6	0.048
M	4.17	0.007	1.25	0.828
Stage	2.29	<0.001	1.52	0.46
IHC	19.46	<0.001	21.45	<0.001

*Note*: *p* < 0.05 considered significant.

Abbreviation: OR, Odd Ratio.

## 4 DISCUSSION

Although still controversial, there is increasing evidence that TFE3‐rearranged RCC is a more aggressive tumor. Adult TFE3‐rearranged RCC is generally diagnosed as advanced disease, progresses rapidly, and has a median survival of only 18 months.^23^ The lymph node metastasis rate of children with TFE3‐rearranged RCC is much higher than that of adult TFE3‐rearranged RCC at the time of diagnosis, and the incidence of metastasis can reach about 50%.^24^ In our study total of 229 cases were included, of which 59 cases (25.9%) were positive. In addition, TFE3‐rearranged RCC is more common in advanced tumors, with lymph nodes metastases distant metastases and prevailed at a younger age. Therefore, how to accurately diagnose TFE3‐rearranged RCC and intervene in time is of great significance.

Studies have reported that the imaging examinations may differentiate TFE3‐rearranged RCC from clear cell carcinoma.^25^, ^26^ But there is no recognized imaging method to diagnose TFE3‐rearranged RCC. Meanwhile, many studies have reported that the initial diagnosis of TFE3‐rearranged RCC was misdiagnosed as clear cell renal cell carcinoma or renal papillary cell carcinoma.^18^, ^27^, ^28^

The main feature of TFE3‐rearranged RCC is that the Xp11.2/TFE3 translocation and forms new TFE3 fusion genes with different partner genes and promotes the expression of TFE3. The reported genes fused with TFE3 include PRCC, ASPL, SFPQ, NONO, RBM10, and so on.^29^, ^30^, ^31^, ^32^, ^33^, ^34^ Whether the fusion of different partner genes will lead to different biological behaviors has not been clearly concluded. Initially, it was reported in the literature that the conventional IHC method had a sensitivity of 97.5% and a specificity of 99.6% for the detection of TFE3 protein nuclear immune reaction.^7^ With the deepening of the research, the researchers found that the results of TFE3 IHC are inevitably affected by many factors.^7^, ^35^, ^36^ As a result, the role of IHC has gradually become controversial. In our study, regardless of the scoring system used for IHC evaluation, it shows good accuracy and sensitivity with the balanced accuracy being 0.802, 0.813, and 0.815, respectively. IHC has confirmed its value in the diagnosis of RCC.

As a prediction tool with high accuracy and good distinguishing features, nomogram is easy to use and promote. Although the diagnosis criteria based on the IHC results are somewhat inadequate, the use of nomograms containing IHC results to predict TFE3‐rearranged RCC is a new concept. In our study, we included four comprehensive and readily available clinical variables to construct nomograms. Its predictions performed well, as evidenced by AUC values of 0.935, 0.934, 0.933, and 0.916 in the training, internal test, all of 208 patients, and external validation sets, respectively. Calibration curves demonstrated that the predictions were consistent with the actual observations. A further diagnosis by FISH based on positive staining for TFE3 by IHC is a standard procedure for the diagnosis of TFE3‐rearranged RCC. However, in our model, age, gender, IHC, and lymph node metastasis were key risk factors for the TFE3‐rearranged RCC. For some IHC‐negative findings, a further diagnosis by FISH should also be performed if there is a high suspicion of TFE3‐rearranged RCC based on other clinical risk factors.

Our research has certain limitations. First, as a retrospective study, internal bias is unavoidable. Secondly, in the selection of cases, we mainly focus on those under 30 years old, which may lead to missed diagnosis of potential cases. Finally, although FISH is clinically the most reliable diagnostic tool for TFE3‐rearranged RCC, there is still the possibility of misdiagnosis. Advantages of this study include population‐based case collection, the use of three different scoring systems, accuracy of risk models, and validation of external data set.

## 5 CONCLUSION

Altogether, our findings confirmed the important role of IHC in the diagnosis of TFE3‐rearranged RCC. Age, gender, lymph node metastasis, and IHC were the most significant predictors of TFE3‐rearranged RCC. The risk model constructed by these four factors improves the accuracy of the diagnosis.

## AUTHOR CONTRIBUTIONS

**Pengju Li:** Data curation (equal); formal analysis (equal); investigation (equal); project administration (equal). **Quanhui Xu:** Data curation (equal); formal analysis (equal); investigation (equal); project administration (equal). **Minyu Chen:** Data curation (equal); investigation (equal). **Jiangquan Zhu:** Data curation (equal); formal analysis (equal). **Yinghan Wang:** Formal analysis (supporting). **Mukhtar A. Mumin:** Writing – review and editing (equal). **Kangbo Huang:** Writing – review and editing (equal). **Zeying Jiang:** Formal analysis (supporting). **Hui Liang:** Investigation (supporting). **Qiong Deng:** Investigation (equal). **Zhu Wang:** Investigation (equal). **Bing Liao:** Investigation (equal). **Wenfang Chen:** Investigation (equal). **Yun Cao:** Resources (equal). **Jiazheng Cao:** Resources (equal). **Junhang Luo:** Project administration (lead); resources (lead); supervision (lead).

## FUNDING INFORMATION

This study was supported by grants from National Natural Science Foundation of China (Award Number: 81725016, 81872094, 81772718, 81602219, 81972376), Guangdong Provincial Science and Technology Foundation of China (Award Number: 2017B020227004, 2017A030313538).

## CONFLICT OF INTEREST STATEMENT

The authors declare that the research was conducted in the absence of any commercial or financial relationships that could be construed as a potential conflict of interest.

## ETHICS STATEMENT

This study was conducted in accordance with the ethical standards of the Declaration of Helsinki and was approved by the ethics review committee of the First Affiliated Hospital of Sun Yat‐sen University and the Cancer Center Affiliated to Sun Yat‐sen University, which waived the need for informed consent in ethics approval.

## Supporting information


Figures S1–S3.

## Data Availability

All data is available in the Article, Supplementary Information files or available from the authors upon reasonable request.
