# Supplementary material for: A nomogram based on TFE3 IHC results and clinical factors as a preliminary screening scheme for TFE3‐rearranged renal cell carcinoma
Source: Cancer Med. 2024 Mar 13;13(5):e6813. doi: 10.1002/cam4.6813 (PMC10935875; doi:10.1002/cam4.6813)
Supplement: Supplementary file 1 — Figures S1–S3. [file CAM4-13-e6813-s001.docx]

**Supplementary materials**


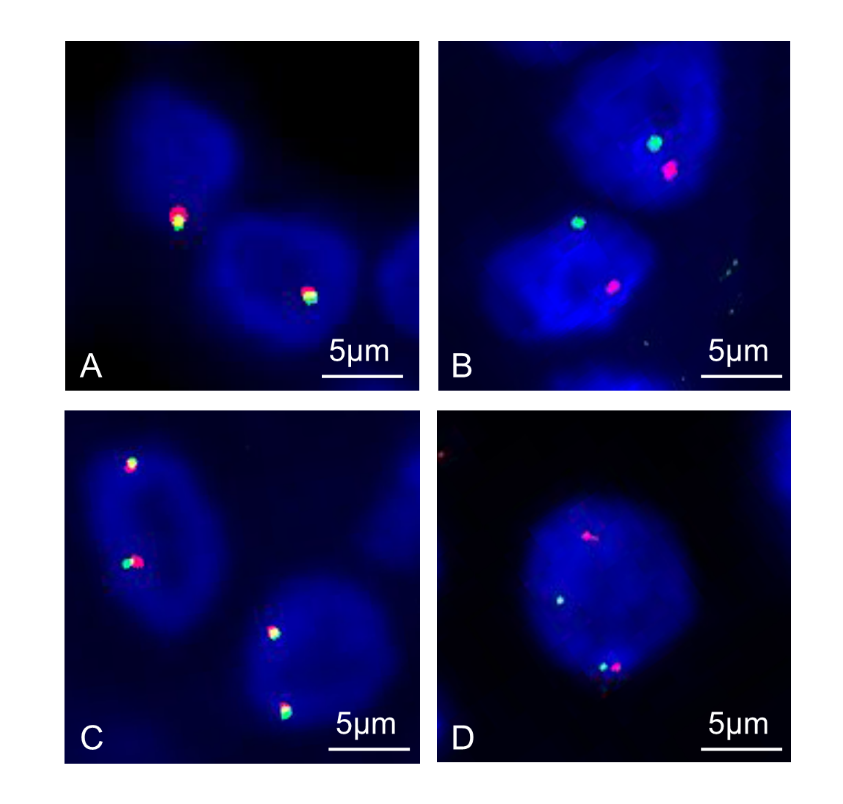


**Supplementary Fig. 1: Representative images of the TFE3 break-apart probe assay.**

(**A-B**) In male patients, a normal result (**A**) exhibited as a fused or closely approximated green-red signal while a positive result (**B**) exhibited as a split-signal pattern.

(**C-D**) In female patients, a normal result (**C**) exhibited as a pair of fused signal while a positive result (**D**) exhibited as a pair of split-signal pattern.


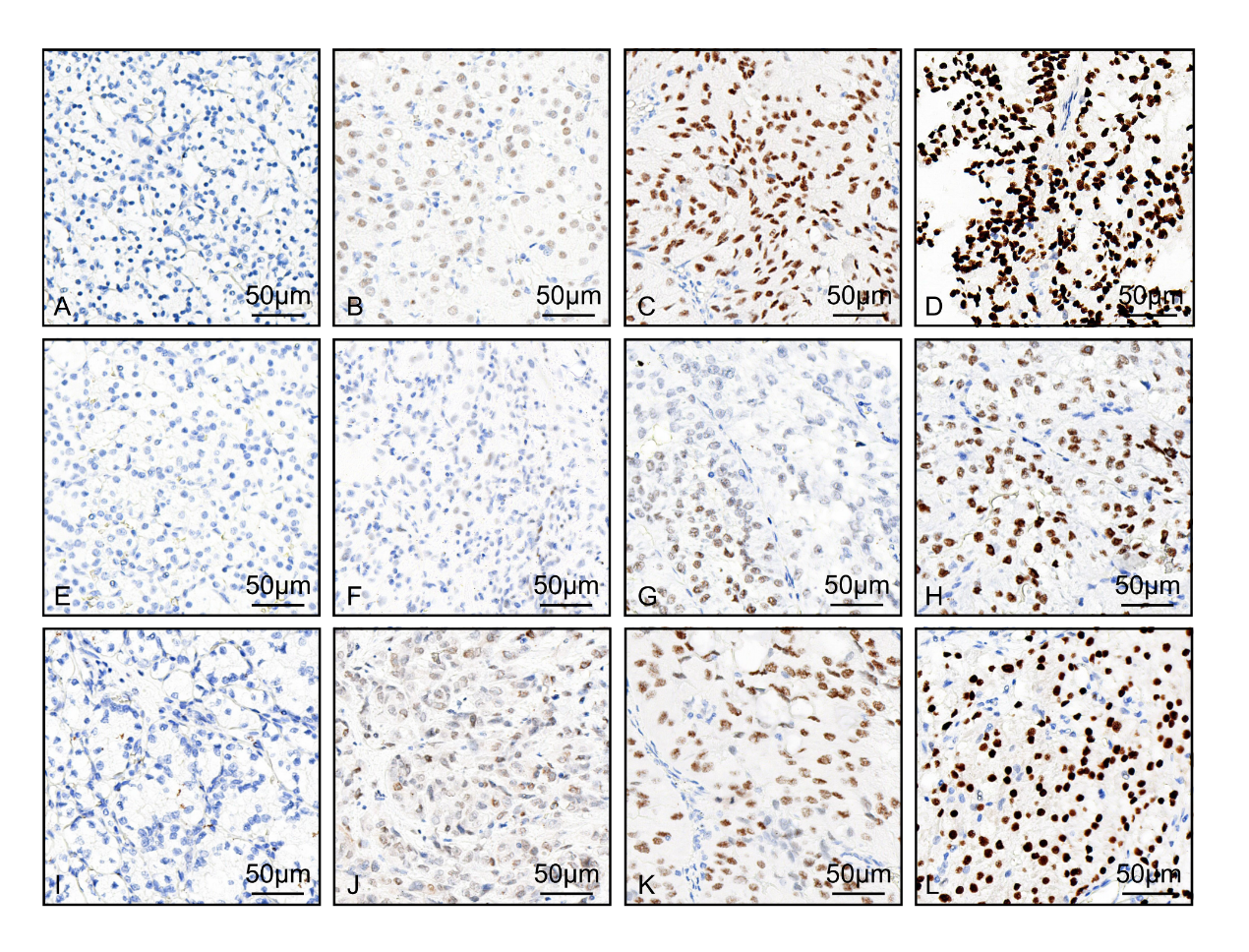


**Supplementary Fig. 2: Representative immunohistochemistry staining for TFE3 based on three scoring systems.**

(**A-D**) The intensity of nuclear staining graded as “0” (**A**), “1+” (**B**), “2+” (**C**) and “3+” (**D**) are shown based scoring system 1. Only (**C**) and (**D**) considered to be positive.

(**E-H**) The percentage of positive tumor cell nuclei scored as “0” (**E**), “1+” (**F**), “2+” (**G**), and “3+” (**H**) based on scoring system 2. Only (**G**) and (**H**) considered to be positive.

(**I-L**) The immunostaining result scored as “0” (**I**), “1+” (**J**), “2+” (**K**), “3+” (**L**) based on scoring system 3. Only (**J**), (**K**) and (**L**) considered to be positive.


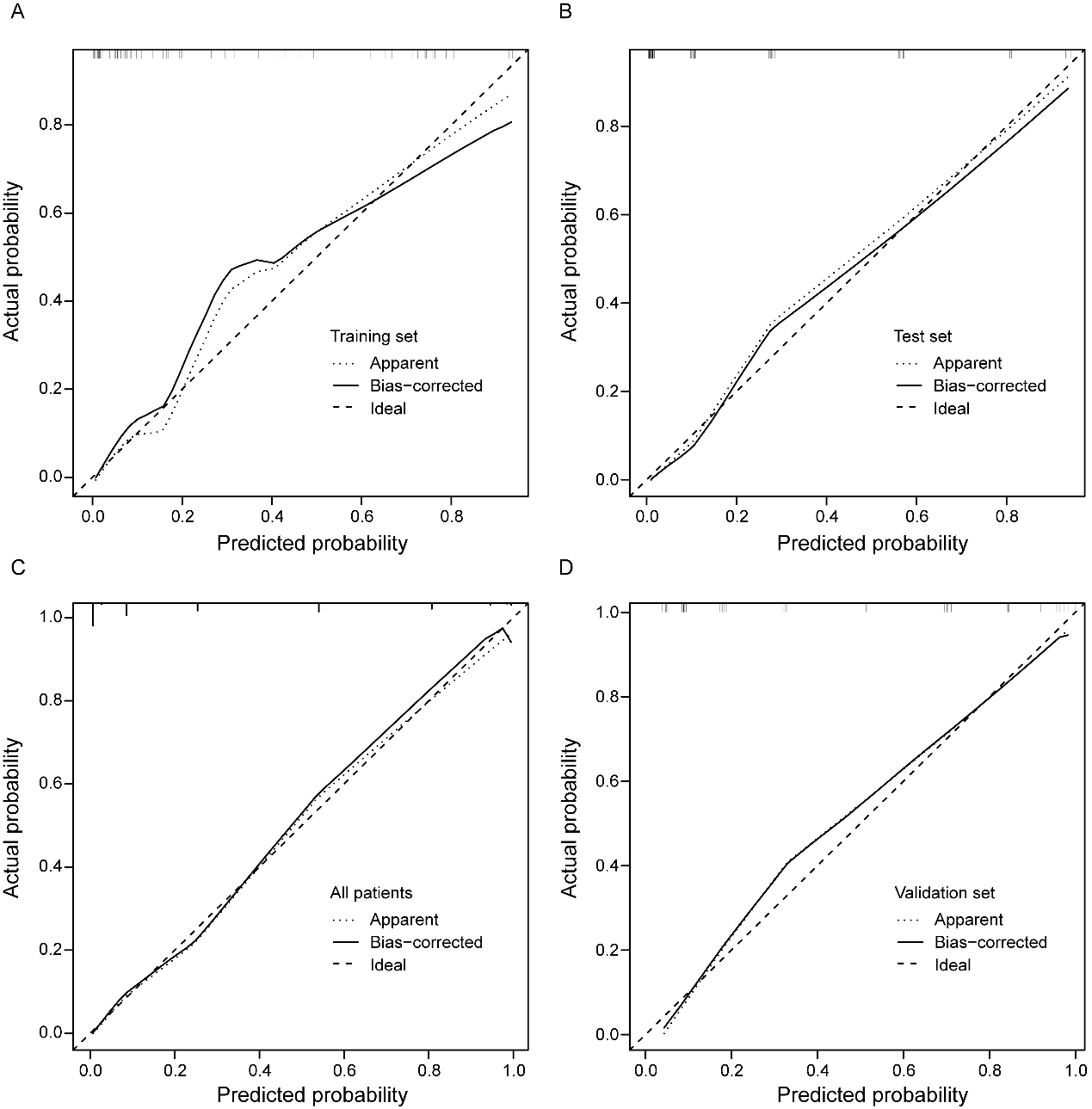


**Supplementary Fig. 3: Plots of calibration results of the nomogram**

Calibration results of the nomogram in the training (**A**), internal test (**B**), all patients (**C**) and external validation cohorts (**D**).
